# Effectiveness and determinants of smoking cessation in the Saudi Arabian Region of Jazan: A cross-sectional study

**DOI:** 10.18332/tid/156842

**Published:** 2023-01-21

**Authors:** Osama Albasheer, Abdulaziz H. Alhazmi, Abdullah Alharbi, Anwar M. Makeen, Ahmad Y. Alqassim, Hafiz I. Al-Musawa, Amjad E. Alabah, Alwaleed K. Alhazmi, Nawaf A. Khormi, Yazeed A. Hamzi, Eyad Y. Abu Sharhah, Riyadh M. Salami, Mohammed Alshareef, Hassan Suwaydi, Ahmed Elkhobby

**Affiliations:** 1 Family and Community Medicine Department, Faculty of Medicine, Jazan University, Jazan City, Saudi Arabia; 2 Emerging and Epidemic Infectious Diseases Research Unit, Medical Research Center, Jazan University, Jazan City, Saudi Arabia; 3 Microbiology and Parasitology Department, College of Medicine, Jazan University, Jazan City, Saudi Arabia; 4 Faculty of Medicine, Jazan University, Jazan City, Saudi Arabia; 5 College of Pharmacy, Jazan University, Jazan City, Saudi Arabia; 6 Family Medicine and Primary Health Care, Ministry of Health, Jazan City, Saudi Arabia

**Keywords:** smoking cessation, determinants of smoking cessation, smoking abstinence program, Jazan, Saudi Arabia

## Abstract

**INTRODUCTION:**

Smoking cessation has significant health benefits. The purpose of this study is to assess the efficacy and related factors of smoking cessation therapies in the Jazan Region of Saudi Arabia.

**METHODS:**

This is a cross-sectional study that took place at smoking cessation clinics in primary healthcare centers between January 2019 and January 2020.

**RESULTS:**

This study enrolled a total of 103 people. The success rate for quitting smoking was 36% at three months, with a 13% relapse rate at six months. Age (p=0.017), occupation (p=0.046), daily cigarette intake (p=0.015), and number of visits (p=0.001) were all found to be significant determinants of smoking cessation. In the multivariate analysis, only the number of visits increased the likelihood to quit smoking (AOR=0.31; 95% CI: 0.15–0.63). Self-efficacy was cited as the primary reason for quitting smoking by 71% of the participants, whereas family support, smoking cessation therapies, and friends’ support were cited as predictive variables by 18%, 10%, and 1% of the participants, respectively.

**CONCLUSIONS:**

Smokers who received the smoking cessation intervention package were three times more likely to succeed in giving up smoking when compared to those who received the routine service. Regular follow-up during smoking cessation interventions significantly enhanced the quit rate. It is recommended that pharmacotherapy strategies and intense therapy performed face-to-face with a cessation counselor be combined to improve the quit rate.

## INTRODUCTION

Globally, tobacco use accounts for 12% of all deaths (approximately 5 million) of adults aged >30 years^1^. The World Health Organization (WHO) reported that deaths from tobacco-related cardiovascular diseases presumably occur among young persons. It is an epidemic with a negative impact on smokers and their family members^2^. Tobacco smoking is a major preventable risk factor for the development of cardiovascular and respiratory diseases. In 55 publications containing 141 cohort studies, the pooled relative risk for coronary heart disease for 20 cigarettes per day was 2.04 in men and 2.84 in women^3^. Even with one cigarette per day, the risk is still high (the pooled relative risk for coronary heart disease was 1.48 in men and 1.57 in women for one cigarette per day)^3^. In addition, smoking increases the threat of different forms of cancers, including lung, colorectal, and liver cancer^3,4^. In Saudi Arabia, the smoking rate of men and women aged ≥15 years was 14.30% in 2020^5^.

Many behavioral interventions are available to enhance smoking cessation (SC) in adults^6^. Behavioral interventions include in-person counseling, telephone counseling, motivational interviewing, and acceptance and commitment therapy^6,7^. They play an important role in the prevention of smoking-related diseases and deaths^8^, and people who quit smoking have lower risks of disease consequences than smokers^9^. Moreover, SC contributes to low morbidity and mortality within all age groups. A study showed that the risk of mortality was reduced by 90% in smokers aged <40 years who quit smoking^10^. Among cancer patients, cessation improves the quality of life, enhances the prognosis, and reduces the risk of recurrence^11^.

In addition to therapy, the WHO has been recommending the provision of cessation services such as clinics and quitlines to facilitate quitting among smokers and reduce the burden of smoking on the health system^12,13^. Smoking cessation clinics have a crucial role in smoking abstinence. Most current smokers report that they would like to quit, and the majority tried to quit during the previous year^14^. Unfortunately, nearly all attempts to stop smoking fail, and after quitting, with or without assistance, it is possible to start smoking again. Many previous studies indicate the risk of relapses increases for younger age, higher severity of nicotine dependence, prior quit attempts, and poor health status^15-18^. In addition, high socio-economic status individuals are more likely to try quitting^19^, and consultations with medical staff improve the rate of abstinence. A study showed that a higher frequency of consultation will result in a higher success rate^20^. A report from Saudi Arabia showed that women who quit smoking were found to have been worried about their health^21^.

The Ministry of Health in Saudi Arabia established smoking cessation clinics as a part of the National Tobacco Control Program with the purpose of decreasing the prevalence of smoking within all groups of the community^22^.

Therefore, the main objectives of this study were to evaluate the effectiveness of the smoking cessation interventions in the Jazan Region of Saudi Arabia and to determine the related factors of smoking cessation. Other objectives were to estimate the abstinence rate among the study participants and to analyze the association between the rate of abstinence and sociodemographic factors.

## METHODS

### Study design and setting

A cross-sectional study was conducted from January 2019 to January 2020 at smoking cessation clinics in the primary healthcare centers of the Jazan Region. Smoking cessation services were available at sixteen primary healthcare centers in that region. The services provided were in the form of counseling and pharmacological management.

### Participants

A stratified random sample was used in this study. The Epi Info program formula required for sample size calculation was used. The minimum recommended size (n) of this study was 146, based on the assumption that (E=8% margin of error), a confidence level of 95%, and population of people in smoking cessation clinics per year is N=5000 (according to the records of the smoking cessation program). As no previous study was found in the region, we assumed a prevalence of quitting at 50%. The participants in this study were smokers who visited the selected smoking cessation clinics from January 2019 to January 2020. Six smoking cessation clinics were randomly selected in the current study, and the participants were randomly selected from each of these clinics. First, we split the Jazan Region into mountain, land, and coast sub-areas, then two smoking cessation clinics were selected randomly from each of the sub-areas. The participants included were male and female adult smokers who received smoking cessation services for at least three months, had regular follow-up, and completed records in the smoking cessation clinics in the primary healthcare centers of the Jazan Region. Confidentiality of information provided by the participants was ensured.

Participants that were excluded included those with critical illnesses such as cancer, those who did not complete their records, and those who did not follow-up on a regular basis. Smokers are defined, according to WHO, as those who smoke regularly or accumulatively for six months or more in their lifetime^23^.

### Data collection tools

The data were collected by an extraction sheet from the participants who visited the clinics. Demographic data were collected including age, gender, education level, occupation, marital status, and ill health or lifestyle risk behaviors such as excessive alcohol intake, poor diet, and physical inactivity^24,25^. These behaviors are linked to hypertension, high cholesterol, and diabetes; they are important contributors to morbidity and mortality^25^. Other data collected include history of chronic diseases, type of smoking, smoking cessation history, and results of the Fagerström test for nicotine dependence to estimate each patient’s physical dependence on nicotine and the predictors of smoking cessation.

After initial consultations, trained investigators conducted assessments at intervals of one week, one month, three months, and six months. Participants were considered successful quitters when they reported they had not smoked within the past month from the start of intervention^26^. Participants who visited the clinic one time and who did not complete one month from the start of intervention were provided follow-up for re-evaluation of their smoking status.

### Statistical analysis

SPSS version 20 (SPSS Inc, Chicago, IL, USA) was used for data analysis. Patient characteristics, abstinence rates, and reasons for failure presented as frequencies and percentages for the risk ratio and incidence rate calculations. A comparison of patients who succeeded in quitting smoking and those who did not was carried out by using χ^2^ and t-tests for the following variables: gender, education level, marital status, Fagerström scores, age at the onset of smoking, a history of previous attempts to quit smoking, and the patient’s perception of his/her level of motivation to quit smoking. A significance criterion of p<0.05 was used in the analysis.

## RESULTS

### Baseline characteristics of the participants and smoking-cessation related factors

Table 1 presents the baseline characteristics of the participants and smoking-cessation related factors. The study sample consisted of 103 participants. Of the study sample, 98% of the participants were male, 74% were married, 74% were governmental employees, and 55% had secondary school diplomas.

**Table 1:** Baseline characteristics of the participants and smoking cessation related factors at smoking cessation clinics in primary healthcare centers between January 2019 and January 2020, Jazan Region of Saudi Arabia (N=103)

*Characteristics*	*n*	*%*
**Age** (years), mean ± SD	38.349 ± 11.199	
**Sex**		
Male	101	98
Female	2	2
**Education level**		
Primary	4	4
Intermediate	11	11
Secondary	57	55
University and postgraduate	31	30
**Occupation**		
Government employee	76	74
Private sector	22	21
None	5	5
**Marital status**		
Married	76	74
Single	25	24
Divorce	2	2
**Ill health behavior**		
Khat	55	53
None	48	47
**Chronic diseases**		
Presence	21	20
Absence	82	80
**Date of starting smoking cessation** (years)		
1	50	49
2	51	50
3	2	2
**Smoking abstinence**		
Yes	37	36
No	66	64
**Smoking cessation related factors**		
Self-efficacy	73	71
Family support	19	18
Friends’ support	1	1
Smoking cessation interventions	10	10

The mean age of the participants was 34.3 years. Additionally, most participants (53%) reported khat chewing. Regarding chronic diseases, 21% reported having chronic diseases. For the date of starting smoking cessation, 74% had started one year prior, 24% had started two years prior, and 2% had started three years prior. Smoking abstinence was found in 36% at three months, while 64% were still smokers. The most reported factor for smoking cessation was self-efficacy (71%), followed by family support (18%). Smoking cessation interventions and friends’ support were reported in 10% and 1% of the participants, respectively.

### Interventions and their consequences

Table 2 presents the smoking cessation interventions and related consequences. The recorded types of smoking among the studied participants were cigarettes (89%), pipes (8%), and shamma (3%). Among the current smokers, 6% of the participants smoked 10 cigarettes or less per day, the highest proportion of participants (39%) smoked 11–20 cigarettes per day, 25% of the participants smoked 21–30 cigarettes per day, and the remaining participants smoked ≥31 cigarettes per day; 98% of the participants were subjected to pharmacotherapy interventions. Nicotine replacement therapy (NRT) was prescribed to 39% of the participants, varenicline was prescribed to 45% of the participants, and the remaining participants used both medications; 13% of the participants relapsed after six months of quitting smoking. Most of the participants (63%) visited the clinic only once during the follow-up, 29% visited it twice, 6% had three visits, and only 2% had four or more visits. The Fagerström test score average was 4.379 (SD=2.513). A total of 98% of participants had previously made quit attempts, as follows: 21% of the participants had one previous quit attempt, 24% had two previous quit attempts, 21% had three previous quit attempts, 19% had four previous quit attempts, and 12% had five or more quit attempts; 2% of the participants had no previous quit attempts.

**Table 2:** Smoking cessation interventions and related factors with their consequences at smoking cessation clinics in primary healthcare centers between January 2019 and January 2020, Jazan Region, Saudi Arabia

*Intervention*	*n*	*%*
**Period from intervention**		
6 months	52	50
1 year	35	34
2 years	16	16
**Type of intervention**		
Psychotherapy	2	2
Pharmacotherapy	101	98
**Prescribed medications**		
Nicotine replacement therapy	40	39
Varenicline	46	45
Combined	17	17
**Number of quit attempts**		
0	2	2
1	22	21
2	25	24
3	22	21
4	20	19
≥5	12	12
**Fagerström score,** mean ± SD	4.379 ± 2.513	
**Number of visits during the follow-up**		
1	65	63
2	30	29
3	6	6
4	1	1
6	1	1
**Relapse**		
Yes	13	13
No	90	87
**Type of smoking**		
Cigarettes	92	89
Pipe	8	8
Shamma	3	3
**Daily cigarette consumption**		
≤10	6	6
11–20	40	39
21–30	26	25
≥31	20	19

### Univariate analysis and multivariate analysis of participants who stopped smoking compared to those who still smoked

Table 3 presents a univariate and multivariate analysis comparing participants who stopped smoking to those who are still smokers and who showed no association in smoking cessation related to sex (p=0.127), education level (p=0.230), and marital status (p=0.222). In contrast, age in the current study had an association with smoking cessation success (p=0.017), as those aged ≤35 years demonstrated a higher rate of smoking abstinence success. In terms of occupation, governmental employees demonstrated a lower percentage of smoking abstinence success than private-sector employees (p=0.046). Additionally, participants with a lower average daily cigarette consumption were more likely to have smoking abstinence success (p=0.015). The number of visits during the follow-up were associated with smoking abstinence (p=0.001), while the number of quit attempts was statistically not significant (p=0.577). In multivariate analysis, only the number of visits seemed to be significantly associated with smoking abstinence; more visits increased the likelihood to quit smoking (AOR=0.31; 95% CI: 0.15–0.63).

**Table 3:** Univariate and multivariate analysis of participants who stopped smoking compared to those who still smoke in smoking cessation clinics in primary healthcare centers between January 2019 and January 2020, Jazan Region, Saudi Arabia

*Variable*	*Univariate analysis^a^*					*Multivariate analysis^b^*	
	*Yes (n=37; 36%)*		*No (n=66; 62%)*		*p*	*AOR*	*95 % CI*
	*Mean*	*SD*	*Mean*	*SD*			
**Age** (years)	35	8	40	12	0.017*	1.04	0.98–1.10
	***n***	***%***	***n***	***%***			
**Sex**							
Male	35	95	66	100	0.127		
Female	2	5	0	0			
**Education level**							
Primary	1	3	3	5	0.230		
Intermediate	1	3	10	15			
Secondary	22	59	35	53			
University and postgraduate	13	35	18	27			
**Occupation**							
Government employee	23	62	53	80	0.046*	4.56	0.29–71.46
Private sector	10	27	12	18		2.63	0.15–47.03
None	4	11	1	2			
**Marital status**							
Married	25	68	51	77	0.222		
Single	12	32	13	20			
Divorced	0	0	2	3			
**Prescribed medication**							
NRT	14	38	18	27	0.775		
Varenicline	18	49	28	42			
Combined	5	14	46	70			
**Chronic diseases**							
Presence	6	16	15	23	0.301		
Absence	31	84	51	77			
	***Mean***	***SD***	***Mean***	***SD***			
**Smoking status**							
Fagerström score	3.9	2.4	4.6	2.5	0.192		
Daily cigarette consumption	2.3	0.74	2.8	0.93	0.015*	1.72	0.84–3.49
Number of visits	1.8	1.04	1.2	0.59	0.001*	0.31**	0.15–0.63
Number of quit attempts	2.6	1.88	2.8	1.57	0.577		

NRT: nicotine replacement therapy. SD: standard deviation. AOR: adjusted odds ratio. The alpha criterion for p-value was set to 0.05.

* Significant in univariate analysis.

** Significant in multivariate analysis.

a Chi-squared test and t-test were used for univariate analysis.

b Multiple logistic regression was used when variable significantly associated with smoking abstinence in univariate analysis.

## DISCUSSION

The current study examined the effectiveness of smoking cessation interventions and determined the abstinence-related factors. The success rate of smoking cessation was 36% at three months for participants who received the smoking cessation intervention package. The number of visits seemed to be significantly associated with smoking abstinence, while other variables like age, occupation, and daily cigarette consumption, though important in smoking cessation, did not reach significant relationships in multivariate analysis.

Most of the study sample were male (98%), which is similar to the findings of several previous studies^27-29^. Giovino et al.^30^ gathered data from 13 countries and reported that the men’s smoking rate was 48.6% compared with 11.3% in women. This gender difference in smoking intake could be related to the low smoking rate among women observed worldwide.

The abstinence rate among the participants in this study was 36% at three months of intervention. This was higher than the generally reported rate of smoking abstinence, which is <10%^31^. The difference could be related to the use of interventions and the interval between the initiation of the intervention and the time of assessment. In Thailand, the success rate was 25.62% at six months of intervention^32^; in China, the success rate was 28.4% at three months of intervention^29^.

In the current study, the univariate analysis of sociodemographic data showed a statistical difference between age and the rate of smoking cessation. Data related to age as a factor in smoking cessation are contradictory. Some studies support that age has an association with smoking cessation success, while others found the opposite^33,34^. Similarly, García-Rodríguez et al.^35^ found that age is a factor in smoking cessation, while Hubert et al.^36^ found that age is associated with smoking behavior. The current study showed that increased age is associated with smoking cessation failure; however, previous studies observed the success of smoking cessation was among older adults^37,38^. Elderly people may be more concerned with their health, especially in the presence of comorbid diseases, and this could explain their high success rate. However, age as a factor for smoking cessation success needs more investigation.

Another aspect of quitting smoking is one’s occupation. There was a statistical difference in this study between the rate of quitting and the kind of occupation (p=0.046). When compared to private-sector employees, government employees had a lower rate of smoking cessation. This finding contradicted a prior study conducted in China that found no link between occupation and smoking cessation^29^. This could be due to discrepancies in how the occupation is classified and the nature of the work.

In previous studies, marital status and education level were significant predictors in stopping smoking^39-41^. A study conducted to evaluate the behavior and attitudes of Saudi nationals demonstrated good attitudes toward anti-smoking campaigns among highly educated people (university degree and higher) compared with low education level (secondary and lower)^39^. Zhuang et al.^40^ reported that the more educated were more likely to quit smoking than the less educated. Additionally, married men had a higher percentage of smoking cessation success than single men^41^. However, the current study found that education level and marital status had no statistically significant relationship with smoking cessation success. Substantial qualitative and quantitative investigations are required to test this association.

In line with a previous study conducted in Taiwan^33^, the current investigation found that the rate of smoking abstinence was substantially associated with daily cigarette intake and the number of follow-up visits. Multivariate analysis reflected the significant importance of the number of visits. Zhu et al.^42^ found that the number of follow-up visits had a significant influence on the success of smoking cessation. Huang et al.^33^ looked at smoking cessation rates in relation to smoking cessation settings and average daily cigarette consumption. In their study, Hu et al.^29^ found that smoking duration, daily smoke consumption, Fagerström score, and exhaled CO value at first visit, were not linked to successful smoking cessation.

In terms of previous quit attempts, the current study found no significant association between successful smoking cessation and previous quit attempts in both univariate and multivariate analysis. A Chinese study found a connection between quit attempts and successful smoking cessation, with higher rates of success associated with more quit attempts^29^. More prior attempts, in theory, show self-efficacy and more exposure to smoking cessation therapies, resulting in a high success rate. In terms of abstinence, however, other aspects such as concurrent drug usage and khat chewing must be considered; 53% of the individuals in this study used khat, a powerful stimulant^43^. The negative correlation in this study could be explained by concurrent khat chewing and smoking. However, the disparities between this study’s findings and those of earlier research require further investigation.

The majority of participants (71%) cited self-efficacy, whereas family support, smoking cessation therapies, and friends’ support were cited as related factors by 18%, 10%, and 1% of the participants, respectively. Similarly, self-efficacy and social support were found to have a significant influence on smoking cessation success in previous studies^43,44^. In addition, family and social support, as well as self-efficacy and smoking cessation interventions, were found to be strong predictors for stopping smoking in Saudi Arabia^27^.

### Limitations

There were some limitations to this research. The limited sample size is the main weakness, which stems from the inability to reach participants by mobile phone and their infrequent attendance at smoking clinics during the study period. Inability to contact participants via phone, especially those with irregular or incomplete follow-up, precludes the detailed assessment of relapse. Furthermore, due to the study design and limited sample size, the temporal relationship between age at the beginning of smoking, marital status, and occupation, and their relations to smoking cessation, cannot be ascertained. Large quantitative and qualitative studies are needed to test these relationships. Finally, because this was a cross-sectional study, the findings cannot be applied to the entire population of Saudi Arabia.

## CONCLUSIONS

In the Jazan Region, smoking cessation clinics had a significant impact on the smoking abstinence rate. Participants who received the smoking cessation intervention package were about three times more likely to succeed in quitting smoking when compared to those who received the routine service. Smokers who received regular follow-up during the smoking cessation interventions exhibited an improved SC rate when compared with the rates of other smokers. The age at beginning of smoking, marital status, occupation, and rate of smoking intake were significant determinants of smoking cessation. It is recommended that pharmacotherapy strategies and intense therapy be performed face-to-face with a cessation counselor to improve the quit rate.

## Data Availability

The data supporting this research are available from the authors on reasonable request.
