# Corrigendum: Effectiveness and determinants of smoking cessation in the Saudi Arabian Region of Jazan: A cross-sectional study

**DOI:** 10.18332/tid/162787

**Published:** 2023-04-06

**Authors:** Osama Albasheer, Abdulaziz H. Alhazmi, Abdullah Alharbi, Anwar M. Makeen, Ahmad Y. Alqassim, Hafiz I. Al-Musawa, Amjad E. Alabah, Alwaleed K. Alhazmi, Nawaf A. Khormi, Yazeed A. Hamzi, Eyad Y. Abu Sharhah, Riyadh M. Salami, Mohammed Alshareef, Hassan Suwaydi, Ahmed Elkhobby

**Affiliations:** 1 Family and Community Medicine Department, Faculty of Medicine, Jazan University, Jazan City, Saudi Arabia; 2 Emerging and Epidemic Infectious Diseases Research Unit, Medical Research Center, Jazan University, Jazan City, Saudi Arabia; 3 Microbiology and Parasitology Department, College of Medicine, Jazan University, Jazan City, Saudi Arabia; 4 Faculty of Medicine, Jazan University, Jazan City, Saudi Arabia; 5 College of Pharmacy, Jazan University, Jazan City, Saudi Arabia; 6 Family Medicine and Primary Health Care, Ministry of Health, Jazan City, Saudi Arabia

**Keywords:** smoking cessation, determinants of smoking cessation, smoking abstinence program, Jazan, Saudi Arabia

Corrigendum on:

Effectiveness and determinants of smoking cessation in the Saudi Arabian Region of Jazan: A cross-sectional study

By Osama Albasheer, Abdulaziz H. Alhazmi, Abdullah Alharbi, Anwar M. Makeen, Ahmad Y. Alqassim, Hafiz I. Al-Musawa, Amjad E. Alabah, Alwaleed K. Alhazmi, Nawaf A. Khormi, Yazeed A. Hamzi, Eyad Y. Abu Sharhah, Riyadh M. Salami, Mohammed Alshareef, Hassan Suwaydi, Ahmed Elkhobby

Tobacco Induced Diseases, Volume 21, Issue January, Pages 1–9,

Publish date: 21 January 2023

DOI: https://doi.org/10.18332/tid/156842

In the originally published version of the article the funding statement was not provided. The statement is now corrected as follows:

“This research is supported and funded by the Deanship of Scientific Research, Jazan University (Support Number: RUP2-06).”

The section has been corrected also online.

